# Postpartum Coronary Vasospasm with Literature Review

**DOI:** 10.1155/2014/523023

**Published:** 2014-07-07

**Authors:** Jayanth Koneru, Matthew Cholankeril, Kunal Patel, Fadi Alattar, Ashraf Alqaqa, Hirtaj Virk, Fayez Shamoon, Mahesh Bikkina

**Affiliations:** ^1^Seton Hall Cardiology Fellowship Program, St. Joseph's Medical Center, Paterson, NJ 07503, USA; ^2^Seton Hall Internal Medicine Residency Program, St. Michael's Medical Center, Newark, NJ 07102, USA

## Abstract

Acute myocardial infarction during pregnancy or the postpartum period is rare. We report a case of a 39-year-old postpartum woman who developed non-ST-elevation myocardial infarction due to severe diffuse coronary vasospasm. To our knowledge, this is the first case of angiographically evidenced coronary vasospasm, in a postpartum woman, with resistance to intracoronary nitroglycerin.

## 1. Case Report

We present a 38-year-old gravida 1 para 1 African American female, status post-C-section two weeks before, who presents with retrosternal, 7/10 severity, and pressure-like chest pain associated with nausea and vomiting for a few hours. The pain was described as indigestion, inducing gradual in onset, multiple episodes, intermittent, and nonradiating chest pain and associated with shortness of breath. No aggravating or alleviating factors were described. The patient had no significant past medical history except for hypothyroidism and no family history of premature coronary artery disease. The patient was a one pack per day smoker and quit ten years before. There was no history of drug use or alcohol use.

**Figure 1:**
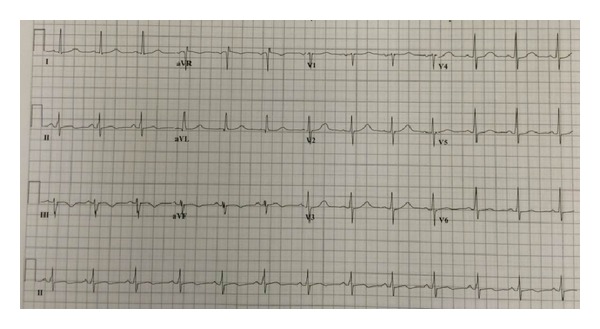


Patient's vital signs were stable with blood pressure 143/94, heart rate 85, body temperature 97.7, and respiratory rate of 20. In the emergency room, she had a computerized tomography scan of the chest, which was negative for pulmonary embolus and no obvious aortic dissection. Significant labs included first Troponin-I reported thirty minutes after initial emergency room contact at 18 ng/mL, creatinine kinase-1227, and a second Troponin-I of 36 ng/mL. The electrocardiogram (EKG) showed a normal sinus rhythm with T-wave inversions in the inferior leads (Figure 1). The patient continued to have pain, but the pain did subside after nitroglycerin drip. As the pain improved, the patient was transferred to the Cardiac Care Unit (CCU), chest pain free, with a provisional diagnosis of Non-ST-elevation myocardial infarction.

**Figure 2:**
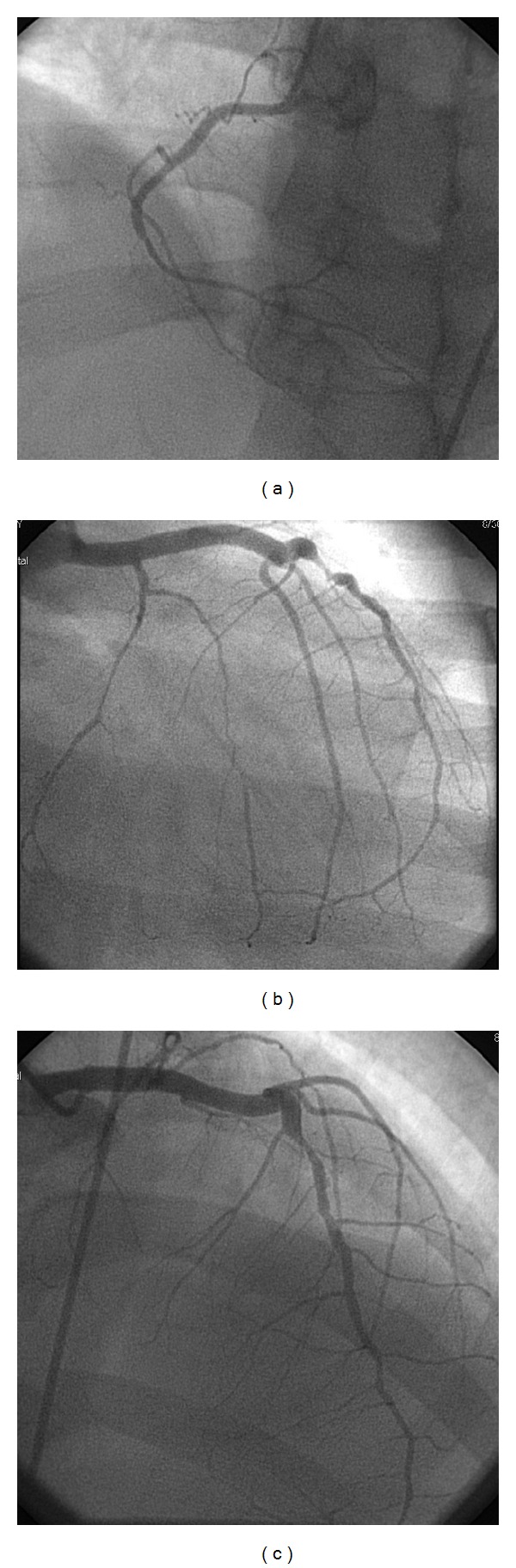
(a) Narrowing of the right coronary artery (RCA) is observed. (b) Narrowing of the left anterior descending (LAD) and left circumflex arteries is observed. (c) Narrowing of the LAD is observed.

**Figure 3:**
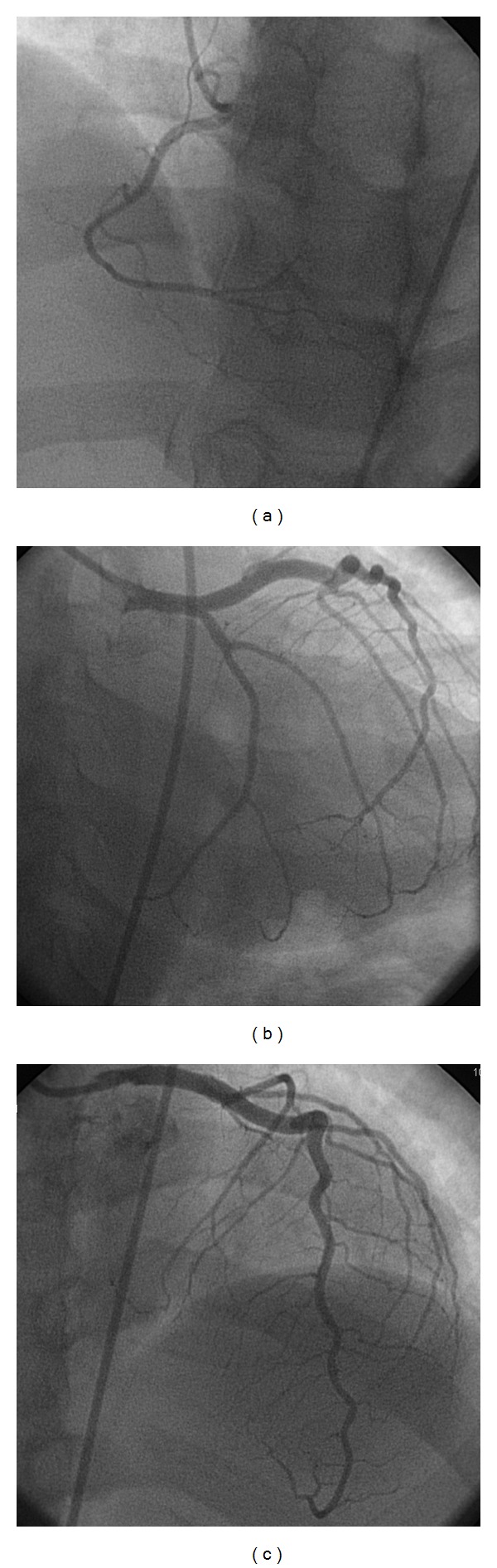
(a) Normal RCA is observed. (b) Normal LAD and left circumflex arteries are observed. (c) Normal LAD is observed.

The differential pathophysiology was debated by the admitting cardiologist, and due to the postpartum period, the potential diagnosis included coronary dissection, atherosclerotic plaque rupture, and Takotsubo cardiomyopathy in the postpartum period. Patient was started on aspirin, plavix, statin, lovenox, and nitrates. A 2D echocardiogram and serial EKGs were ordered. The 2D echocardiogram was reported as mildly reduced left ventricular systolic function of 45% with severe inferior and inferolateral hypokinetic regional wall motion abnormality. Thereafter, the patient underwent cardiac catheterization the subsequent day (Figures 2(a), 2(b), and 2(c)). Based on catheterization, which showed severe triple vessel diseases even after delivering eight intracoronary injections of nitroglycerine at 50 mcg each, the interventionalist decided to perform FFR of the midleft anterior descending (LAD) artery lesion which showed 0.78, without adenosine injection. It was concluded at this juncture that a second opinion was warranted regarding percutaneous coronary intervention (PCI) versus coronary artery bypass graft (CABG) especially with small caliber vessels and right coronary artery (RCA) likely being the culprit for the symptoms with normal TIMI 3 Flow. Patient opted for PCI and was scheduled after the postpartum period (two months later) to decrease risk of bleeding from antiplatelets given for PCI. The second catheterization (Figures 3(a), 3(b), and 3(c)) showed normal coronaries.

This shows a clear case of vasospasm induced myocardial ischemia not relieved by intracoronary nitroglycerin. This case is unique as the only one other case in literature was described having coronary vasospasm postpartum and even more unique that intracoronary nitroglycerin failed to relieve vasospasm, which has never described in a postpartum patient to our knowledge.

## 2. Discussion

### 2.1. Pathophysiology of Coronary Vasospasm

Coronary spasm was first reported by Prinzmetal et al., who showed reversible myocardial ischemia accompanied by ST-segment elevation on the EKG [1]. Coronary artery spasm is defined as a dynamic, transient reduction in the luminal diameter of the epicardial coronary arteries due to increased vasomotor tone leading to myocardial ischemia. It is widely accepted that coronary spasm plays an important role in the pathogenesis of variant (Prinzmetal) angina, sudden cardiac death, and other forms of ischemic heart diseases [2].

The pathogenesis of coronary spasm is complex and not fully understood. It is thought to be a multifactorial process involving several mechanisms. These include endothelial dysfunction, hyperreactivity of coronary smooth muscle cells to constrictor stimuli (catecholamines, acetylcholine, and histamine), and other triggering factors, such as activation of the parasympathetic nervous system and alpha-adrenergic receptors [3]. Endothelial dysfunction is thought to play a primary role in the development of coronary artery spasm. The endothelium is crucial in the regulation of coronary vascular tone, mainly through the release of several vasodilators, the most important of which is nitric oxide (NO). Therefore, it is thought that any impairment of endothelium-mediated vasodilatation might favor the induction of coronary spasm by release of vasoconstrictors at the site of these predisposed segments [4].

Long acting nitrates have long been used in patients with coronary spasm for their vasodilatory effects. Nitroglycerin (NTG) is an endothelium-independent vasoactive agent with the capacity to diminish myocardial oxygen demand by dilating peripheral arteries and veins, thereby causing a resultant fall in left ventricular preload and afterload. It also augments myocardial oxygen supply by dilating epicardial coronary arteries and increasing collateral and subendocardial blood flow [5]. Experimental and clinical studies have demonstrated that the increase in angiographic cross-sectional area of normal coronary arteries after nitroglycerin administration can reach 30% to 50% from that at baseline [6].

The pharmacokinetics of nitroglycerin is still not completely understood. Multiple studies have shown that the measurement of plasma concentrations of nitroglycerin cannot be used to predict their direct hemodynamic effects. Bogaert hypothesized that a likely explanation for this is that when organic nitrates are introduced into the body and start to elicit their effect, mechanisms countering this effect begin to operate. These are counterregulation and tolerance. Counterregulation, that is, reflex adaptation, is well known and has been documented in many studies with organic nitrates. Tolerance, the decreasing ability of the vascular smooth muscle cell to respond to a given concentration of the organic nitrate, has also been well documented in animals and is widely accepted to be present in humans [7].

The attenuation of the effect of nitrates with time, either due to counterregulation or due to tolerance, has led to intensive study of dose regimens and the eventual emergence of the concept of the “nitrate-free period.” Milicevic et al. found that, in 41 of 55 patients, tolerance to NTG could be overridden by multiple dose escalations over a period of up to 48 hours [8]. The interval of time between the onset of tolerance and secondary resistance to nitroglycerin is clinically important because it provides a therapeutic window during which benefits of nitrate effects can be extended by upward dose titration.

Patients who develop coronary artery spasm usually respond to sublingual (SL) and/or intravenous (IV) formulations of nitroglycerin, but occasionally, these routes of administration are not completely effective in controlling manifestations of acute ischemia. It is not known why SL or IV NTG fails to relieve coronary spasm in some patients. One explanation is that spasm reduces blood flow to the vasa vasorum of the stenotic segment and prevents the drug from reaching the area. A second possibility is reflex sympathetic stimulation occurring from NTG induced systemic vasodilation [9].

Some of the most important indications for intracoronary nitroglycerin include spontaneous spasm refractory to conventional methods of nitrate administration or spasm induced during coronary catheterization, angioplasty, or ergonovine testing. Intracoronary NTG ideally allows higher concentrations of NTG at the site of coronary arterial constriction and has a direct relaxant effect on the smooth muscle of the spastic coronary segment [10]. Goto et al. measured coronary perfusion, phasic pattern of inflow to the myocardium, and transmyocardial distribution of blood flow to understand the direct effect of nitroglycerin on coronary hemodynamics. Their study showed that intracoronary administration of nitroglycerin (1) decreased poststenotic coronary arterial pressures, (2) increased the diastolic velocity but more greatly augmented the systolic reverse velocity, and (3) increased the subepicardial blood flow [11].

### 2.2. Postpartum Acute Myocardial Infarction

The uncommonness of pregnancy-related acute myocardial infarction (AMI) makes it a complex diagnosis. However, AMI can occur at any time during pregnancy, delivery, or postpartum and can result in maternal and/or fetal complications including death. The incidence of AMI during pregnancy is increasing as advances in reproductive technology aid many older women to conceive [12]. This presents a unique challenge to cardiologists and obstetricians, who must understand the incidence, risk factors, and etiology of pregnancy-related AMI to formulate the diagnosis and be equipped to manage the patient's medical and obstetrical needs adequately. The incidence of ischemic heart disease in women of reproductive age is uncommon, but risk of AMI increases by 3- to 4-fold during pregnancy. In particular, pregnancy-related AMI has been reported up to 6.2 cases per 100,000 pregnancies [13]. Data comprised of case reports from various institutions and a population-based study have not been described. In a case series presented by Roth and Elkayam, 208 cases of pregnancy-related AMI were reviewed in which 78% had undergone coronary angiogram and only 42% of the women had significant coronary artery stenosis. Coronary vasospasm as the cause of AMI was identified in only three patients, accounting for only 1.8% of all pregnancy-related AMI patients [14]. de Vuyst et al. have published the only other known case postpartum coronary vasospasm, but their patient's vasospasm was relieved by nitroglycerine [15].

It is known that vasoconstrictive agents such as ergonovine, bromocriptine, and prostaglandin E2, which are frequently used during pregnancies, can induce coronary vasospasm [16–19]. The diagnosis of coronary vasospasm was presumed in most of the cases that were reported in literature, as signs of myocardial injury were noted on laboratory evaluation or by imaging with the presence of insignificant coronary artery disease. Other pathophysiological mechanisms of coronary vasospasm in pregnancy may be due to increased renin release and angiotensin production from uterine hypoperfusion [20]. Some other hypotheses of vasospasm in pregnancy that have been considered include endothelial dysfunction, as well as angiotensin II and norepinephrine effects [21–23].

### 2.3. Takotsubo Cardiomyopathy

Takotsubo cardiomyopathy in the postpartum period should also be considered in the differential diagnosis of chest pain in the postpartum patient. This cardiomyopathy can be precipitated by intense emotional or physical stress or another medical illness. It is characterized by transient systolic dysfunction, typically of the apical and sometimes midsegments of the left ventricle, and it can present similarly to an AMI [24]. The pathophysiology of this diagnosis is still being investigated, and one theory is that this cardiomyopathy is caused by an excess of catecholamines leading to microvascular spasm or dysfunction and potentially myocardial stunning [25]. As there can be the occasional finding of coronary vasospasm in Takotsubo cardiomyopathy [26], there is overlap between the diagnosis of Takotsubo cardiomyopathy and coronary vasospasm. Although typically women are more prone to this cardiomyopathy than men [27], usually the women are in the postmenopausal age group [28], in contrast to this patient's age. Typically the diagnosis is considered when the patient has EKG changes that are more severe than the elevation in cardiac biomarkers [29]. This patient had a more serious elevation in cardiac biomarkers than is usually seen in Takotsubo cardiomyopathy. Also the patient lacked the findings usually seen on echocardiography such as typical apical ballooning or midventricular hypokinesis. A cardiac MRI showing absent late gadolinium enhancement may support the diagnosis of Takotsubo cardiomyopathy [30]; however, a cardiac MRI was not performed due to low suspicion of Takotsubo cardiomyopathy in this patient.

### 2.4. Multivessel Coronary Vasospasm

Coronary artery vasospasm is caused by the constriction of the smooth muscle in the coronary arteries which can lead to variant angina pectoris in many patients. Patients with coronary spasm have endothelial dysfunction and are suffering from a low-grade chronic inflammation [31]. Although it typically involves one coronary artery, there have been case reports of multivessel coronary spasm [32]. The incidence of multivessel spasm has not been frequently reported; however, diffuse-multivessel spasm has been associated with patients with vasospastic angina. Park et al. attempted to demonstrate the incidence of diffuse multivessel spasm by dividing patients into three groups, one of which had diffuse-multivessel spasm patients and in whom it was seen that these patients have higher rates of male sex, alcohol drinkers, and mean triglyceride, with lower levels of high density lipoprotein [33]. It has been reported that the incidence of coronary artery spasm is higher in Korean and Japanese individuals, compared to that of Western people; however, the exact frequency of multivessel spasm is unknown, except that it is rare, and diffuse-multivessel spasm is more rare [34]. Coronary vasospasm in general is associated with other vasospastic conditions such as neurologic disorders, that is, migraine, and rheumatological conditions, that is, Raynaud's phenomenon. Patients also tend to be younger compared to patients with coronary disease, and exercise has been shown to induce symptoms, especially in the early morning [35]. Coronary artery vasospasm has been associated with, and may be caused by, atherosclerotic plaque formation [36].

In addition to coronary vasospasm being associated with sites of coronary atheroma, patients will have an impaired response to vasodilator therapy. This is because the vascular endothelium secretes both vasorelaxing factors and vasoconstrictive agents, and patients with vasospasm have dysfunction of the endothelium. This can lead to vasoconstriction and can lead to the typical presentation of coronary vasospasm patients, which is variant angina. The difference in mechanism between diffuse-multivessel spasm and localized vasospasm is unclear; however, it has been observed that diffuse three-vessel coronary artery spasm usually occurs spontaneously [34]. Typically the diagnosis should be considered on the differential when patients present with angina associated with EKG changes, with the pain and EKG changes resolving either spontaneously or with nitroglycerin [37]. Coronary spasm incidence is decreasing likely secondary to using medications that indirectly decrease coronary spasm as well as decreased smoking which may be a trigger [38, 39]. As a result, it is less frequently diagnosed and recognized even when it occurs as fewer clinicians are aware of it [40]. The attacks of coronary spasm are associated with either ST segment elevation, ST segment depression, or negative U wave on EKG [41]. Patients with multivessel coronary spasm may have unstable arrhythmias and can be resistant to standard medical therapy such as Ca-channel blockers. Precipitating factors can include exertion, medications such as catecholamines, parasympathomimetics, and histamine, and many others [42]. Coronary vasospasm can potentially lead to coronary thrombosis. Definitive diagnosis is usually made with angiography showing vasoconstriction that reverses with nitroglycerin administered intravenously or intra-arterially [37]. Diagnosis can also be through ambulatory EKG, or the intracoronary injection of acetylcholine (Ach) in doses of 10–100 *μ*g can be used for provocation of coronary spasm [43]. The attack of coronary spasm can usually be promptly treated by the sublingual administration or oral spray of nitroglycerin or isosorbide dinitrate (ISDN) [44]. For the prevention of attack, long-acting drugs are needed and calcium channel blockers are very effective for this purpose [2]. It is possible that RhoA/ROCK pathway blockers may prove to be useful for the treatment of coronary spasm [45].

Case reports have detailed various presentations of multivessel coronary vasospasm. Typically these patients present with coronary occlusion on coronary angiography that is unmasked through intracoronary administration of nitroglycerin. Ho et al. describe a case of a patient presenting with chest pain, EKG findings of ST depression, and elevated cardiac markers, with cardiac catheterization revealing right coronary artery occlusion with diseased, nonobstructive left anterior descending (LAD) and left circumflex (CIRC) arteries. However, after administration of intracoronary nitroglycerin, a normal looking RCA was unmasked, and the LAD and CIRC arteries appeared bigger in calibre and were nonobstructive [46]. Similarly, Krim et al. describe a case whereby a patient with a history of migraine headache, taking sumatriptan, presents with chest pain and shortness of breath. Her EKG showed ST-segment elevation in leads II, III, aVF, and V2 through V6 with further coronary angiography revealing severe stenosis of the proximal LAD artery and the second obtuse marginal artery and a totally occluded mid-LAD artery. Intracoronary administration of nitroglycerin led to resolution of all stenoses and the patient was advised to stop taking sumatriptan [47]. Han et al. presented a case whereby a 50-year-old female presented with chest pain, usually occurring at rest in the morning, and following ergonovine provocation test, diffuse three-vessel coronary artery spasm was identified [48]. In a different presentation, Gowda et al. submitted a case of a 56-year-old male patient that presented with recurrent exertional angina pectoris with multivessel coronary vasospasm seen during cardiac catheterization that was considered partly spontaneous and partly catheter-induced due to the mechanical irritation [49]. Also, Van Spall et al. showed a case of 68-year-old male with no prior history of coronary artery disease presenting with epigastric pain and found to have diffuse ST segment elevation on EkG. The patient's coronary angiography demonstrated 70% proximal LAD stenosis, which later resolved with intracoronary nitroglycerin [37].

## 3. Conclusion

Due to difficulty in angiographically diagnosing coronary vasospasm, it can be easily missed. However, it should be strongly considered as the cause of AMI in the absence of significant coronary stenosis. Simply treating with nitrates or calcium-channel blockers can prevent progression to recurrent refractory spasms associated with increased morbidity and even death.
